# Orthopædics in General Practice

**Published:** 1910-12-17

**Authors:** J. Jackson Clarke

**Affiliations:** Senior Surgeon to the Hampstead and North-West London Hospital, and Surgeon to the Royal National Orthopædic Hospital.


### HOSPITAL CLINICS.

### ORTHOPÆDICS IN GENERAL PRACTICE.

By J. JACKSON CLARKE, M.B. (London), F.R.C.S.; Senior Surgeon to the Hampstead and North-West London Hospital, and Surgeon to the Royal National Orthopædic Hospital.

(An Address delivered at the opening of the Session of the Willesden and District Medical Society, on Friday, October 21, 1910. Dr. Percy Evans, President, in the Chair.)

MR. PRESIDENT AND GENTLEMEN,—The first particular study of a group of the affections of childhood was, I believe, that formulated in an interesting little book, entitled "l'Orthopédie," by Andry, in 1751. Andry was physician to the French Court, and he defined "orthopædy" as "the art of preventing and correcting bodily deformities in children." At that time, I believe, there were no special hospitals for children. At the present day orthopædics include the treatment of congenital deformities, the hygiene and physical training of children, the prevention and the effects of rickets, tuberculosis, infantile paralysis, rheumatism, rheumatoid arthritis, and other nutritional and infectious disorders. In its proper meaning orthopædics thus belong to the family physician as much as to the surgeon. The surgical side of orthopædics is now applied to the treatment of deformities, disabilities, and diseases affecting the locomotor organs, no matter at what age. It is thus a wide subject, but there are certain parts of it which come oftener to our notice than others, and on these I beg your indulgence while I speak.

Of congenital deformities I will mention only one, the common club-foot. In every instance, with proper treatment, a perfect result can be obtained. Adams's method is probably still the best of all, and in this method a period of simple splinting and manipulation, which can well be done under the supervision of the family medical man, precedes the operative measures.

### STATIC DEFORMITIES.

There are a certain number of deformities which are named static, including flat-foot, knock-knee, and many cases of lateral curvature or scoliosis. These static deformities have, especially in German books, been explained under Wolff's Law, which runs: "Every change in the static relations of a bone not only leads to a corresponding change of internal structure, but also to a change of external form and physiological function." Wolff was Professor of Orthopædic Surgery in the University of Berlin. In his view, genu valgum is a physiological accommodation to abduction of the legs; scoliosis to a twisted posture of the vertebral column. Thus Wolff regarded these deformities as being purely mechanical affections. I do not think anyone in England regards them in that light. The mechanical factor certainly is important, but I think we all agree that the pathological factor is still more important. This view I stated in 1899 *—namely, that the pathological factor is the most important one in the causation of the so-called static deformities. Physiological accommodation takes place in the altered structure of the bones, and this makes the deformity permanent when it is allowed to exist long enough for such changes to occur. We know that bones alter in internal structure rapidly; we can see it in the course of repair of a broken bone with angular union. We know that the part of the callus which is most nearly in a straight line between the ends of the bone becomes developed, and the rest, even the projecting angle of the original bone, atrophies. This is a simple example of how far Wolff's law applies. If the deformities were caused in a simple mechanical way, we should expect to cure them in a simple mechanical way. We may imagine an adult who has genu valgum dating from childhood: the bones have become hard and set in the deformed shape. If one should try to cure him by making him stand cross-legged he would have to stand so for a very long time, and even then he might not be cured. And his parting words to his medical man might well be, "*Ars longa, vita brevis.*" Rickets plays a large part in the development of deformities. There is another result of indigestion which we often see in children; I call it the gouty form. It is marked by excess of urates in the renal excretion, by irritability of muscles and nerves, skin eruptions, furred tongue, constipation, and so forth. Children affected in this way are very apt to get deformities akin to those one sees in rachitic children. The muscles are affected more frequently than other structures; there are contractures of the calf muscles, leading to slight talipes equinus; pes cavus and retracted toes, hammer-toes, flat-foot, Achillodynia are also common; scoliosis is common in this type also, but generally it appears at a later age than the rachitic form. The rachitic and gouty types of deformity often coexist in the same patient. Thus in the prevention of deformities attention to slight disorders of nutrition in children is very important. The gouty and rheumatic deformities that have their origin in later life—*e.g.* Dupuytren's contraction—can also be prevented in most cases by the use of simple exercises and instruments.

### KNOCK-KNEE.

We may take knock-knee as an example of deformity requiring orthopædic treatment. We should first try to get it well by restoring the muscular tone which is lost and leads to the adoption of the abduction posture. Weakness of muscles is the chief factor, more even than altered structure and softening of bone. With proper diet and by massage and plenty of fresh air we should try to restore muscular tone. We may find these measures are not sufficient, and then we should order some special exercises. For knock-knee

* *Orthopædic Surgery*: A Text-book of the Pathology and Treatment of Deformities.

there is nothing better than the "outside-edge exercise": the knees straight, the feet parallel and a few inches apart, the patient rises smartly upon the outer edges of the feet. This may be repeated from ten to thirty times. When the patient has had some practice this exercise can be done with crossed legs: right leg in front for half the number of times, then left leg in front for the remainder. We might find that attention to diet and exercise is not sufficient, and then the next factor would be to apply splints at night when the muscles are at rest; by limiting the use of an instrument to the night we avoid depriving the child of the daily exercise of the muscles, and in many cases the addition of the night splint is sufficient. We should know from the first examination of the patient whether there was any fixity about the deformity. In such a case we have to apply instruments for use by day, and the day instrument for knock-knee is that which I now show you—the Thomas's genu valgum splint. The example of it I showed you just now was not quite complete; it will be made complete by the addition of a bar at the back of the knee. There is an important fact with regard to genu valgum: no instrument that allows the knee to bend can assist in removing the deformity.

### Operative Treatment of Knock-knee.

Last of all comes the question of operation. It is very seldom that I have to do a Macewen's osteotomy, and I never do it in children under ten years. In most cases I wait until the patient reaches the age of fifteen or sixteen. Until that age we can generally correct the deformity by means of instruments alone; I will not say in every case, but it has been so in every one I have met with during the last five or six years. I operated upon one last week in the hospital in a youth of nineteen, and in this patient the bones had become hard. I think that whilst growth is going on, and especially whilst the rickety process, which is the cause of the deformity, is active, it is wrong to do Macewen's operation, because if splints such as I have shown you to-night are not worn after the operation for two years the deformity will recur, and the same splints which are necessary after operation will cure the deformity without any operation at all. It is only where the bones have become hard and fixed that the operation is necessary. In the converse deformity, pronounced bow-legs, I have operated upon a considerable number of cases, and have had very gratifying results, by taking out a wedge with its base outwards from below the great trochanter, wiring the ends of the bone together, and so correcting the deformity. The patients who are sent to one for operation, whether it is for genu valgum or genu varum, are those who have not had the attention of the family doctor during the progress of the deformity. One complex static deformity, extreme rigid flat-foot, can be corrected by well-considered surgical operations; such feet have to be converted into the equinus form before the final arthrodesis of the astragalo-navicular joint is done. As to scoliosis, I might repeat about that all that I have said about genu valgum. We try hygienic treatment and exercises; the latter require to be carefully supervised and so planned that they exercise all the muscles of the body, especially the respiratory ones. The best respiratory exercises for spinal affections for delicate children are done with the patient lying down. They rest the spine and teach the child at the same time how to breathe. Adenoids is a subject which is always coming up in connection with chest deformities and scoliosis. The causation of adenoids, in my opinion—and they occur so frequently in orthopædic cases that I have studied them pathologically and clinically for years—is exactly the same as the causation of these diseases I have mentioned; it is either part of rickets or part of the gouty disturbance of the physiology of the body. Of course I do not say children have tophi, etc., but it is the same pathological disturbance which leads to the formation of such deposits in later life.

### Lateral Curvature.

The early stages of scoliosis afford a field for general treatment; suitable exercises alternating with rest and hygienic measures nearly always succeed, but if anyone sets out to make a special study of scoliosis in the hope that they will cure every case, I am afraid that they will be grievously disappointed. Here is a photograph of a very curved spine taken from a child whom I have had under observation since she was a baby; she is now seven years old. Hers is an obstinate case, and she has not been improved by all my efforts, though I have tried exercises and forcible correction under anæsthesia. This excellent skiagram shows the reason of this: four vertebræ are fused together, forming a wedge-shaped portion of the spine. The case is one of congenital scoliosis. Some day we may get as far as to do an osteotomy of the spine, and to take out a wedge of the body of a vertebra; but we have not got to that yet, and until we do such cases are quite incurable. Sprengel's deformity, congenital elevation of the shoulder, also comes into the same class of case, because there are great deformities of the bodies of the vertebræ in pronounced Sprengel's deformity. Cases of scoliosis due to infantile paralysis, syringomyelia, and Friedreich's disease are also very intractable. One patient with Sprengel's deformity who was sent up to me from the country had been kept lying down for two years to cure the scoliosis, of course without any result at all.

### Foot Deformities.

Reverting to the gouty type of deformity, some of them are easily overlooked, and it is only by doing a series of movements, which I hope to show you later, that one finds out when the hamstrings are contracted, or whether the calf muscles are contracted, in which case the ankle cannot be bent with the knees straight to the proper extent—*i.e.* 22° beyond the right angle. In various other ways one finds whether there is disturbance of the neuromuscular system, and it is in some of these cases that there is a condition almost akin to the *forme fruste* of Friedreich's disease: some of these subjects are almost ataxic, the irritability of their neuro-muscular system is so great. And many lives are made quite unhappy by the neglect of this condition when found in childhood: it leads to many deformities—pes cavus, retracted toes, and hammer-toes, to sinking of the arches of the foot. Not only in childhood but in adult life the transverse arch sinks, and there is consequent metatarsalgia. The same type of disorder of nutrition leads to easy deformation of the foot by boots, hallux valgus, etc., and a great deal of suffering and of almost sub-conscious discomfort is caused by these small deviations of the normal form of the foot. Corns are frequently caused by some slight deformity of the foot, and they are especially likely to form in persons of the rheumatic or gouty type—I mean the kind of rheumatism which is made worse by drinking too much beer and eating too much starch or sugar. I need only mention the corns over the heads of the second, third, or fourth metatarsal bones, which indicate a flattened transverse arch and so often lead to metatarsalgia.

### Instruments and Apparatus.

I have shown you some instruments useful in static and paralytic deformity. The instrument question is, of course, a very serious one, and I cannot say we have put it on a right basis. I should like to see in every large hospital an instrument dispensary, and to have an instrument dispenser. I should like to see in every medical school a materia chirurgica and mechanopœia on the lines of the Materia Medica and Pharmacopœia. But this I am sure of, that the principles of the more important and simpler instruments should be taught in the early period of clinical training. Many surgeons still seem to leave the choice and even the design of the instruments to the mechanician. Take the case of one of the most valuable of instruments, Thomas's single hip splint. The surgeon should measure the patient for it, and he should shape it with wrenches before it is covered. The same applies to Chance's spinal splint. Each of the instruments you see here I have designed myself. In difficult cases I have the instruments fitted on in the rough to see if there are any alterations to be made; and I see that they are made of the proper materials and are quite comfortable to the patient as well as efficacious in their action. But it is with instruments as with any other remedial agent which we employ: we must aim at the effective minimum of treatment. The lighter we can make the instruments, and the shorter the time that they are required to be worn, the better for the patient. The mention of these two splints—Chance's for the spine and Thomas's for the hip, the best splints I know for the conditions for which they were designed—brings to mind the subject of tuberculous disease of bones and joints. With proper conservative surgical treatment the results in such cases are so admirable that I have rarely had to have recourse to vaccines which some surgeons have found to be helpful. Neglected cases where secondary infections have occurred give occasion for severe operations, amputations, excisions, and the like. In apparently hopeless cases of spinal disease operative measures, such as laminectomy and costo-transversectomy, have given me most gratifying results.

### Poliomyelitis.

Now I come to another class of cases, infantile paralysis. In the pathology of this disease there has been evolved in the last two or three years important new facts. It is now recognised to be an infective disease, transmissible to animals, and on account of the selective habit of the virus for certain cells of the central nervous system it has been likened to hydrophobia, and hence it is probably of protozoal origin. There is hope that we may soon get to the source of infantile paralysis, but at present we are concerned more with the treatment of it. I will now mention a case which seems to show that judicious and very careful massage may be employed with safety early in paralysis.

Case 1, a boy of six.—On December 9, 1906, he complained of being tired, asking to lie down. On December 13 he was sick after dinner, and was put to bed. He was very hot all night, and complained of bad headache. On the 14th he had bad pains in the back, but his temperature was normal. On December 16 he lost the use of all his limbs; his temperature was 99°, and he was very sleepy. Next day he had a slight return of use in the upper limbs, but was still sleepy. On the 18th he had very acute pains in the back and limbs, and could not bear to be touched. When I saw him on December 19 he was as helpless as a pithed frog; he could only move his head and his eyes a little. The diaphragm was acting irregularly, the pulse was rapid and irregular, and he looked to me as if he might die. I thought his medulla oblongata was partly affected. His mother is a very good masseuse, and in her skill I had the utmost confidence. So in this case I ordered very light massage at an earlier stage than I have known it to be employed in any other case. Another feature in this case was the appearance of patches of red spots on the buttocks, a tiny vesicle forming in the centre of red papules. They looked like an eruption of herpes. The whole attack had been diagnosed as epidemic cerebro-spinal meningitis, or spotted fever. Kernig's sign was absent, and, although the pain was unusual for anterior poliomyelitis, I concluded in favour of the latter diagnosis. The boy wore instruments for a time to correct the little residual drop of the feet, and I do not think anybody would now know that the boy had had infantile paralysis at all.

### A Second Case.

The second case forms a contrast to the foregoing.

Case 2, a girl aged six, who came into the hospital six months ago.—I was puzzled at first to make out what had happened because the legs were absolutely rigid at that time; the knees could not be bent, though now there is some movement in them. Both feet were in a position of pronounced equinus, one with some varus and cavus added, and that was of extreme degree. These deformities I have corrected by operation. I now demonstrate these and other points to you, a nurse having kindly brought the patient from the Orthopædic Hospital. You see she has some scoliosis, and if we ask her to sit up from the supine position we find she cannot rise more than half-way towards the sitting posture—the hamstring muscles are so short that they will not allow the pelvis to assume the position of a right angle with the line of the thighs. This illustrates a useful test to ascertain whether the hamstring muscles are shortened. A person should be able to sit up a little beyond a right angle. This girl has some lateral curvature, and when she first came she had a semi-transparent look, like that of watered milk, which she may have been nourished on in the infirmary in Wales whence she came to us. She has knock-knees, which, as you see, are more pronounced on one side than on the other. I have no doubt this genu valgum developed at the same period as the other deformities when the patient was lying in bed. For her use in the daytime I have had other instruments made, and to correct the knock-knee a second kneecap is added to draw the knee outwards; also a ring-catch by means of which the knee-joint is kept straight in walking. I shall have added at the back of the knees bars like those of Thomas's genu valgum splints. You will see that much more is to be done before this girl can regain her muscular movement. Her cerebral condition is quite sound. At first the stiffness suggested cerebral diplegia with a spastic condition of both limbs, but the case is one of infantile paralysis, in which the cord has been very extensively involved, the child having been kept at rest in bed without massage or other treatment. The rigidity in the limbs of this child is unusual, and can only be explained by the fact that the bones have been growing, whilst the muscles have not been alternately stretched and relaxed by the action of the joints; the muscles have kept their minimum length, while the bones have grown. It is one of many examples that show that genu valgum can form without a child standing up at all. The medical treatment of infantile paralysis, then, includes electrical treatment and massage chiefly, and the massage in the early stages must be limited to the most superficial effleurage, just sufficient to encourage the circulation.

### A Note on the Surgical Treatment.

When we come to the surgical treatment of infantile paralysis we find it is that which gives us most of our work in orthopædic hospitals. The measures we employ extend from simple instrumentation to excisions of joints, arthrodesis, etc. I will not go through all the details. With regard to instruments, lightness is sought after. In Germany celluloid is used a great deal, but in that country they do not usually have open fires. It is so inflammable that I do not use it much here. Wherever it will answer the purpose I use aluminium. I have already emphasised the importance of not using instruments to excess, and it is the same with regard to operations. We hear of new operations from time to time. Lately plastic operations on nerves were much discussed in infantile paralysis. We know that nerve anastomosis between the facial nerve and the spinal accessory or hypoglossal succeeds very often and is a very useful operation indeed, but I have never seen a single case which I should be proud of in which nerve-transplantation has been used for infantile paralysis. For this reason I have not used it in this condition. Tendon transplantation we still use to a considerable extent. I have mentioned arthrodesis, and I do use that operation, but never until the patient is fourteen years of age or more. It is no use doing it when the bones at the joint are largely cartilaginous, but I have had very gratifying results in very obstinate cases, such as extreme paralytic talipes calcaneus, where the patient walks on the heel with the toes in the air. These cases are very difficult to help with instruments, and I have found arthrodesis, when done thoroughly, of very great service.

As I hope you will discuss what I have put before you, I will only say a few words in conclusion, and those in justification of the study of deformities at special cliniques. I am glad to say we have now in London the largest and most modern orthopædic hospital in the kingdom, and, I believe, as good as any in the world, and much work is being done there.

Progress is facilitated where a great number of cases are collected of a particular class. In proof I need only mention the method of treatment for congenital dislocation of the hip devised by Lorenz, Professor of Orthopædic Surgery at the University of Vienna, as a recent advance in surgery. A few years ago surgeons would say that to cure a congenital hip was impossible, but it is now established that three out of every four such dislocations can be cured and the patients enabled to walk as if they have never had anything the matter with them. In the treatment of congenital dislocation of the hip, as in most orthopædic work, there is need of loyal co-operation.

